# Past lake shore dynamics explain present pattern of unidirectional introgression across a habitat barrier

**DOI:** 10.1007/s10750-016-2791-x

**Published:** 2016-05-09

**Authors:** Kristina M. Sefc, Karin Mattersdorfer, Caroline M. Hermann, Stephan Koblmüller

**Affiliations:** Institute of Zoology, University of Graz, Universitätsplatz 2, 8010 Graz, Austria

**Keywords:** Asymmetric introgression, Hybridization, Lake Tanganyika, *Tropheus*, Population structure, Environmental change

## Abstract

Introgression patterns between divergent lineages are often characterized by asymmetry in the direction and among-marker variation in the extent of gene flow, and therefore inform on the mechanisms involved in differentiation and speciation. In the present study, we test the hypothesis that unidirectional introgression between two phenotypically and genetically distinct lineages of the littoral, rock-dwelling cichlid fish *Tropheus moorii* across a wide sandy bay is linked to observed differences in mate preferences between the two lineages. This hypothesis predicts bi-directional nuclear gene flow and was rejected by congruent patterns of introgression in mtDNA, AFLP and microsatellite markers, with admixture confined to the populations west of the bay. This pattern can be explained on the basis of habitat changes in the course of lake level fluctuations, which first facilitated the development of a symmetric admixture zone including the area corresponding to the present sand bay and then shaped asymmetry by causing local extinctions and cessation of gene flow when this area became once more inhabitable. This conforms with previous assumptions that habitat dynamics are a primary determinant of population-level evolution in *Tropheus*. In this respect, *Tropheus* may be representative of species whose preferred habitat is subject to frequent re-structuring.

## Introduction

Patterns of introgression between diverging lineages inform about mechanisms involved in differentiation and speciation. For instance, asymmetries in the direction of gene flow and among loci can elucidate various pre- and postzygotic processes that determine the evolutionary trajectories of the involved lineages. In animals, potential sources of asymmetric introgression include asymmetries in mate choice, driven, for example, by lineage-specific mating preferences and dominance relationships (Rosenfield & Kodric-Brown, 2003; Sefc et al., 2015; While et al., 2015), geographic variation in the fitness of immigrants and hybrids (Nosil et al., 2005; Carson et al., 2012), asymmetric range expansions (Johannesen et al., 2006; but see Zhang, 2014), sex-specific dispersal (Beysard et al., 2012), sex-specific hybrid mortality (Johannesen et al., 2006), and cytonuclear incompatibilities (Arntzen et al., 2009).

**Fig. 1:**
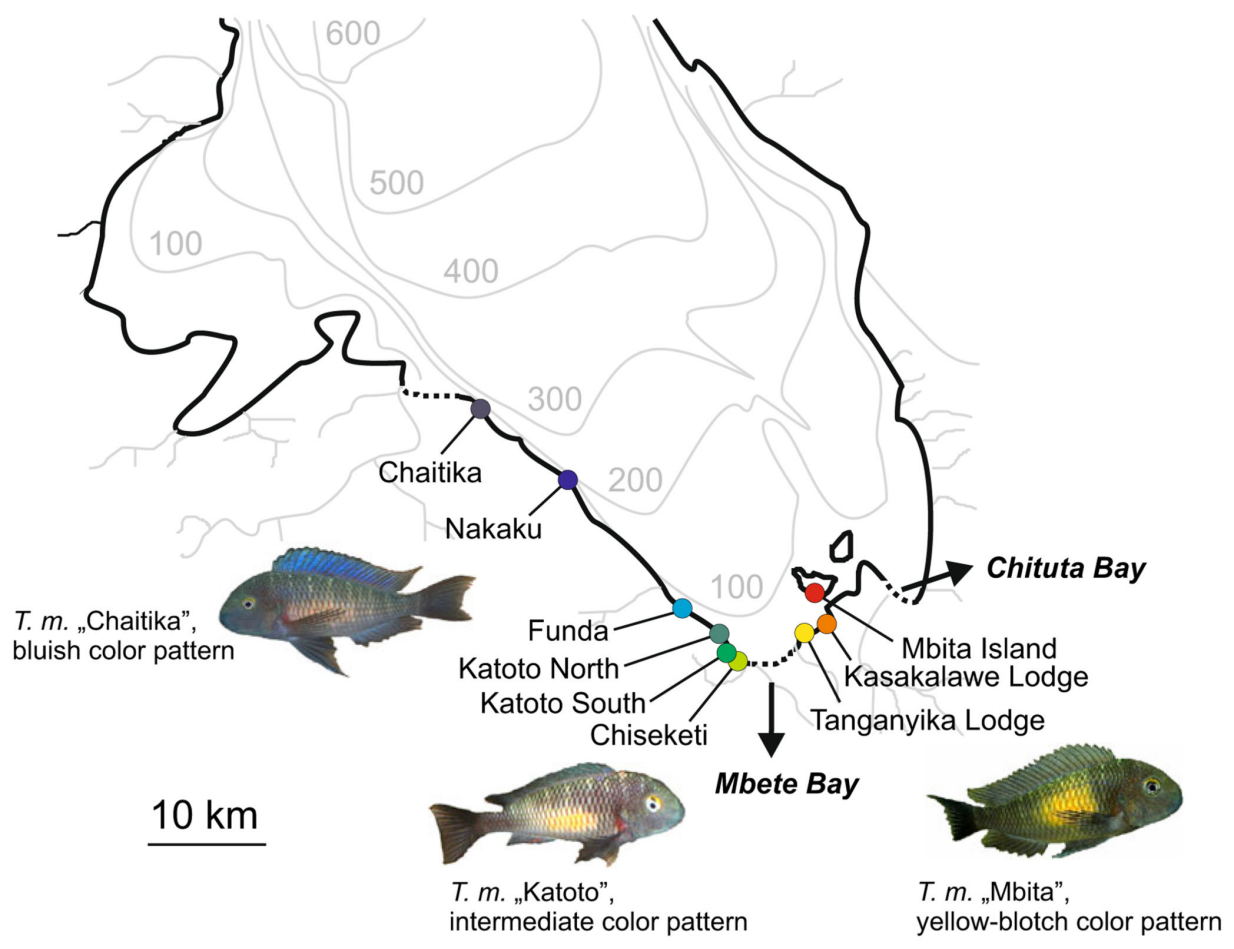
Map of southern Lake Tanganyika, illustrating origin and color pattern of the sampled populations. Stippled sections of the shoreline indicate large stretches of sandy habitat

In the cichlid fish species *Tropheus moorii*, mitochondrial and phenotypic variation suggested unidirectional introgression between two genetically distinct color morphs across a major habitat barrier (Sefc et al., 2007). *Tropheus* are stenotopic inhabitants of the shallow rocky littoral of Lake Tanganyika. High levels of color pattern variation and genetic differentiation among populations are attributed to the presence of numerous dispersal barriers, as the rocky shoreline is frequently interrupted by stretches of sand and by estuaries of rivers and small streams (Egger et al., 2007; Sefc et al., 2007; Koblmüller et al., 2011). Color pattern and genetic differentiation among populations generally increase with geographic distance (which is typically correlated with the number of habitat interruptions), and major habitat barriers often separate phenotypically and genetically highly distinct lineages (Egger et al., 2007). At the very southern tip of Lake Tanganyika, the 6 km wide stretch of sandy shore in Mbete Bay constitutes such a major barrier to rock-dwelling cichlid species (Fig. 1). In all cichlid species investigated so far, populations show high levels of genetic differentiation across Mbete Bay (*Variabilichromis moorii*, Duftner et al., 2006; *Ophthalmotilapia ventralis*, Sefc et al., 2007; *Perissodus microlepis*, Koblmüller et al., 2009) or do not even share mtDNA haplotypes (*Neolamprologus caudopunctatus*, Koblmüller et al., 2007; *Eretmodus cyanostictus*, Sefc et al., 2007). Many species display distinct color pattern differentiation across the bay, and the distribution range of several species ends at Mbete Bay (Kohda et al., 1996). These observations suggest a long-standing interruption of dispersal in that area. In *T. moorii*, Mbete Bay separates the ranges of two distinct color morphs, a rather uniformly colored bluish-gray morph west of the bay from a morph ornamented with a yellow-blotch on the flank east of the bay (Fig. 1). The two morphs correspond to divergent genetic lineages (Egger et al., 2007; Sefc et al., 2007). Although drops of the lake level have altered the structure of the littoral habitat in geological time frames and launched secondary contact among previously separated populations (Sturmbauer et al., 2005; Egger et al., 2007; Koblmüller et al., 2011; Nevado et al., 2013), Mbete Bay has been considered a permanent barrier to rock-dwelling species (Kohda et al., 1996; Koblmüller et al., 2011). Nonetheless, *Tropheus* populations immediately west of Mbete Bay carry signatures of introgression from the eastern yellow-blotch lineages. Their color patterns are intermediate to those of the bluish and the yellow-blotch lineages, featuring a yellow-blotch that is smaller and fainter than that of the pure yellow-blotch morph (Kohda et al., 1996; Konings, 2013). Mitochondrial admixture among the two genetic lineages was demonstrated in one of the intermediate populations (30% yellow blotch-specific mtDNA haplotypes; Sefc et al., 2007). In contrast, yellow-blotch populations east of Mbete Bay showed no evidence of mitochondrial introgression from the bluish lineage (Sefc et al., 2007; Koblmüller et al., 2011). If gene flow across this major barrier was possible at some point, why was it not symmetric?

Several mechanisms that could explain unidirectional gene flow across Mbete Bay appear unlikely given our current knowledge of *Tropheus* biology. Reciprocal experimental crosses between bluish and yellow-blotch *Tropheus* produce fertile male and female offspring (C. Sturmbauer, unpublished data), which argues against cytonuclear incompatibilities and sex-specific (female) hybrid mortality that could otherwise explain the lack of introgressed mitochondrial haplotypes in the populations east of Mbete Bay. Lineage-specific dispersal behavior, i.e., a higher dispersal propensity of yellow-blotch than of bluish *Tropheus*, is rejected by similar and high levels of genetic differentiation among bluish-morph populations west of the bay (Sefc et al., 2007) and among yellow-blotch populations east of the bay (Koblmüller et al., 2011), suggesting similar responses to habitat barriers in both morphs. There is no asymmetry in competitive success in contests between the two color morphs (Sefc et al., 2015). Strong drift in the populations east of Mbete Bay, which could have eliminated western haplotypes, is ruled out by the high genetic diversity in these populations (Koblmüller et al., 2011). Strong selection against immigrants or hybrids east, but not west, of Mbete Bay could produce the observed pattern, but the cause for such location-specific selection remains obscure as habitats and species communities are comparable in the two areas.

A more plausible explanation for unidirectional mitochondrial introgression despite bi-directional migration lies in the mate preferences of the two *Tropheus* morphs. Two-way mate choice experiments and mating patterns in pond-held groups of bluish and yellow-blotch *Tropheus* demonstrated that both bluish and yellow-blotch females strongly prefer bluish over yellow-blotch males (Egger et al., 2008; Sefc et al., 2015). Hence, yellow-blotch females migrating into a bluish population would readily mate with bluish males and introduce their mitochondrial genomes into the sink population, whereas bluish females migrating into a yellow-blotch population may not accept yellow-blotch males and fail to spread their mitochondrial genomes. Bluish male immigrants, in contrast, would enjoy high mating success with resident yellow-blotch females and provide for nuclear gene flow from the bluish into the yellow-blotch lineage. The present study tests this prediction by comparing mitochondrial and nuclear (microsatellite and AFLP) introgression between the bluish and yellow-blotch *Tropheus* lineages across Mbete Bay. If *Tropheus* crossed Mbete Bay in both directions, but mitochondrial introgression east of Mbete Bay was prevented by mate choice behavior, we expect signatures of nuclear admixture on both sides of the bay contrasting with asymmetric mitochondrial introgression. In contrast, if the observed asymmetric mitochondrial introgression was due to unidirectional migration of individuals across Mbete Bay, nuclear introgression would also be unidirectional, and nuclear admixture should be evident in the western populations only.

## Materials and methods

### Sampling

Populations were sampled at nine locations (Fig. 1) between 2003 and 2009 (2003: Kasakalawe Lodge; 2005: Chaitika (10 individuals), Nakaku, Katoto North, Katoto South (27 ind.), Tanganyika Lodge, Mbita; 2008: Funda; 2009: Chaitika (22 ind.), Katoto South (5 ind.), Chiseketi). Fin clips were preserved in >95% ethanol for DNA extraction employing proteinase K digestion and ammonium acetate precipitation (Sambrook & Russell, 2001). Color pattern type and sample size per population are given in Table 1.

### mtDNA sequence analysis

Amplification and sequencing of the mitochondrial control region followed a standard protocol as in Koblmüller et al. (2011). The amplification was carried out in two PCR reactions with primer pairs L-Pro-F_*Tropheus* (Koblmüller et al., 2011)—TDK-D (Lee et al., 1995) and SC-DL (Lee et al., 1995)—TDK-DH4-T (Nevado et al., 2009). Sephadex™ (Amersham) purified Sanger sequencing reaction products (Big Dye Termination Reaction Mix, Applied Biosystems) were run on an ABI 3130xl automatic sequencer (Applied Biosystems). Sequence alignment was done using MUSCLE (Edgar, 2004), and checked by eye in MEGA v.6 (Tamura et al., 2013), and 22 bp of a poly-T region and adjacent positions were removed because of poor signal quality. The final data matrix comprised 797 bp. Sequences are deposited in GenBank under the accession numbers KX184420-KX184714.

Diversity indices were calculated in DnaSP v.5.0 (Librado & Rozas, 2009). Pairwise population differentiation was tested in ARLEQUIN v.3.1 (Excoffier et al., 2005) against corrected *P* values (Benjamini & Hochberg, 1995). Identical sequences were collapsed into haplotypes using DNACollapser in FaBox (Villesen, 2007). jModelTest (Posada, 2008) was used to identify the best fitting model of molecular evolution based on Bayesian Information Criterion (BIC). A neighbor-joining (NJ) tree with 1,000 bootstrap replicates was constructed in MEGA.

### Chronology of population splitting and past population size dynamics

Parameters of divergence time, migration rates, and effective population sizes were inferred under an isolation with migration model (Hey & Nielsen, 2004) by coalescence simulations in IMa2 (Hey & Nielsen, 2007; Hey, 2010). Analyses of pairwise divergence used the following sample units: Chaitika; Nakaku; Mbita Island; Tanganyika Lodge pooled with Kasakalawe Lodge; yellow-blotch lineage individuals from Chiseketi, Katoto South, Katoto North and Funda; bluish lineage individuals from Funda; bluish lineage individuals from Katoto North; and bluish lineage individuals from Katoto South and Chiseketi. Runs were replicated at least two times with different random number seeds. The model employed the HKY model of sequence evolution (Hasegawa et al., 1985). The first 100,000 steps were discarded as burn-in time, and runs were continued until effective sample sizes (ESS) for each estimated parameter were >200 (Kuhner, 2009). To translate parameter estimates into absolute values, we assumed an average generation time of 3 years for *Tropheus* (Egger et al., 2004) and minimum and maximum substitution rates of 0.0178 and 0.0311 per site per MY, respectively, for the whole mitochondrial control region (Nevado et al., 2009). Pairwise divergence times among the above described sample units were illustrated in a NJ tree computed in MEGA. Divergence time between the two major lineages (bluish, yellow-blotch) was inferred by calculating their net divergence in MEGA.

Population size trajectories through time were inferred by means of Bayesian skyline plots (BSPs; Drummond et al., 2005) in BEAST 1.8 (Drummond & Rambaut, 2007). Separate analyses were carried out for the following sample units: individuals from Chaitika and Nakaku; individuals from Tanganyika Lodge and Kasakalawe Lodge; bluish lineage individuals from Funda, Katoto North, Katoto South and Chiseketi; and yellow-blotch lineage individuals from Funda, Katoto North, Katoto South and Chiseketi. MCMC chains were run for at least 5 × 10^6^ generations, with model parameters and trees sampled every 1,000 generations. We employed the HKY+I+G substitution model (selected as best fitting model by jmodeltest) with a strict molecular clock (as we are looking at intraspecific data; Brown & Yang, 2011) assuming the same substitution rates as above, and a Bayesian skyline tree prior (Drummond et al., 2005). The first 10% of generations were discarded as burn-in. Chain convergence to stationarity for all model parameters was assessed in Tracer 1.6 (available from http://beast.bio.ed.ac.uk/tracer). The various datasets required different run lengths, but all analyses were run until post-burn-in ESS for all parameters exceeded 200. The median and corresponding 95% highest posterior density (HPD) intervals were visualized with Tracer 1.6 (available from http://beast.bio.ed.ac.uk/tracer).

### Nuclear marker analysis

AFLP genotyping with 18 selective primer combinations was carried out as described in Mattersdorfer et al. (2012). Negative controls consisting of restriction, ligation, and PCR chemicals and 56 replicate samples were included for error rate estimation and genotype scoring in AFLPSCORE 1.4a (Whitlock et al., 2008). Per-locus mismatch error rate was <1.5%. The final data matrix consisted of 1,160 loci. Indices of genetic diversity and differentiation were calculated in AFLP-SURV version 1.0 (Vekemans, 2002). Significance levels in multiple tests were assessed against corrected *P* values (Benjamini & Hochberg, 1995). Following microsatellite results (see below), Hardy–Weinberg equilibrium was assumed for the estimation of allele frequencies (Zhivotovsky, 1999). Tests for pairwise linkage disequilibrium among AFLP loci were carried out in Arlequin v.3.5 (Excoffier et al., 2005).

The following 16 microsatellite loci were amplified in PCR reactions: UNH2016 (Albertson et al., 2003); UNH908 (Carleton et al., 2002); Pzeb2, Pzeb3 (van Oppen et al., 1997); UNH130, UNH154 (Lee & Kocher, 1996); UME002, UME003 (Parker & Kornfield, 1996); TmoM11, TmoM27 (Zardoya et al., 1996); Hchi1, Hchi6, Hchi36 (Maeda et al., 2007); Pmv3, Pmv17 (Crispo et al., 2007); and Ppun9 (Taylor et al., 2002). PCR products were run on an ABI 3130xl automatic sequencer (Applied Biosystems) and sized against GeneScan-500 ROX internal size standard (Applied Biosystems). Allele size calling was carried out in GENEMAPPER v.3.7 (Applied Biosystems). Tests for Hardy–Weinberg and linkage equilibrium and pairwise population differentiation were calculated in ARLEQUIN v.3.1 (Excoffier et al., 2005). There was no significant deviation from HWE after Bonferroni–Holm correction for multiple testing, and all loci were used for further analyses. *P* values for differentiation tests were corrected with the method of Benjamini & Hochberg (1995).

Both AFLP and microsatellite data were analyzed in STRUCTURE v.2.3.3 (Pritchard et al., 2000) with *K* values ranging from 1 to 10. STRUCTURE output was analyzed with STRUCTURE HARVESTER v.0.6.6 (Evanno et al., 2005; Earl, 2012) to determine the most likely number of genetic clusters. Linkage disequilibrium among AFLP and microsatellite loci was compared among populations as percentage of locus pairs that deviated from equilibrium expectations with *P* < 0.05.

## Results

### Direction of mitochondrial and nuclear introgression

**Fig. 2:**
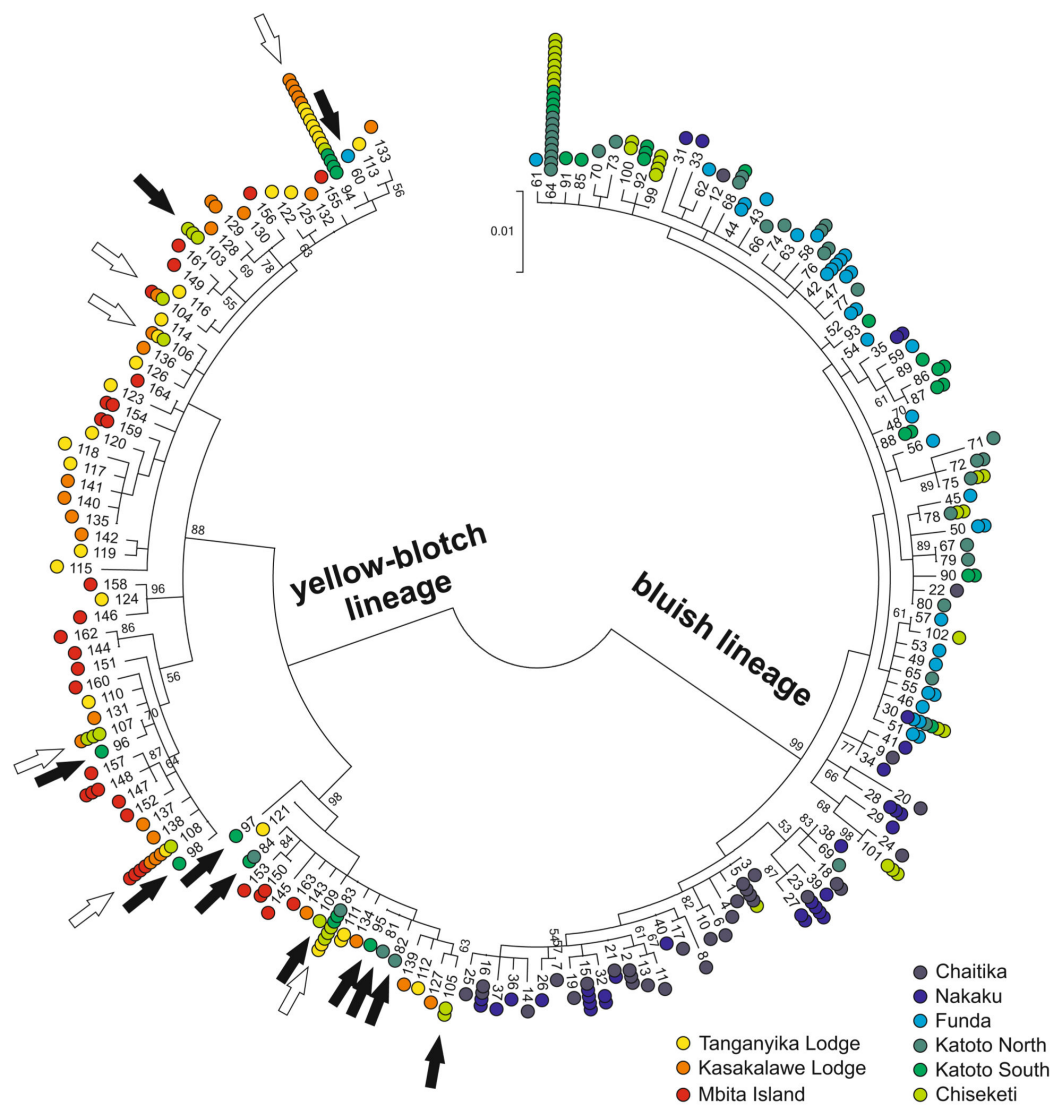
Relationship among mitochondrial control region haplotypes illustrated in a Neighbor-Joining tree. Sample provenience is *color coded*. *Arrows* point out yellow-blotch lineage haplotypes that were detected west of Mbete Bay (*white arrows*, haplotype shared with populations east of the bay; *black arrows*, haplotype private to populations west of the bay). Bootstrap values >50% are indicated next to the respective node

**Fig. 3:**
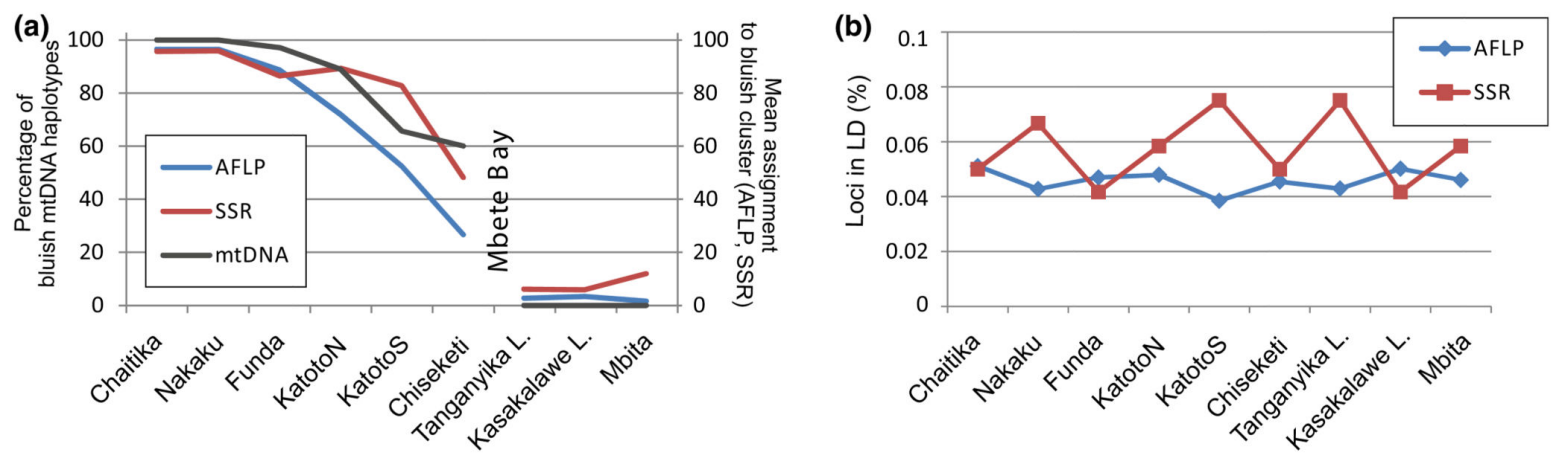
**a** Genetic admixture per population in mitochondrial (percent bluish lineage haplotypes in the population) and in nuclear markers (STRUCTURE assignment probabilities to bluish lineage averaged across individuals within populations, for *K* = 2). **b** Linkage disequilibrium per population, as percent of locus pairs that deviated from equilibrium expectations with *P* < 0.05

Variation in mtDNA haplotypes confirmed unidirectional mitochondrial introgression between populations east and west of Mbete Bay (Fig. 2). In populations west of Mbete Bay, the proportions of introgressed “yellow-blotch lineage” haplotypes ranged from 3% to 40% and decreased gradually with distance from the bay (Fig. 3a). In contrast, no introgressed “bluish lineage” haplotypes were detected in the populations east of Mbete Bay.

**Fig. 4:**
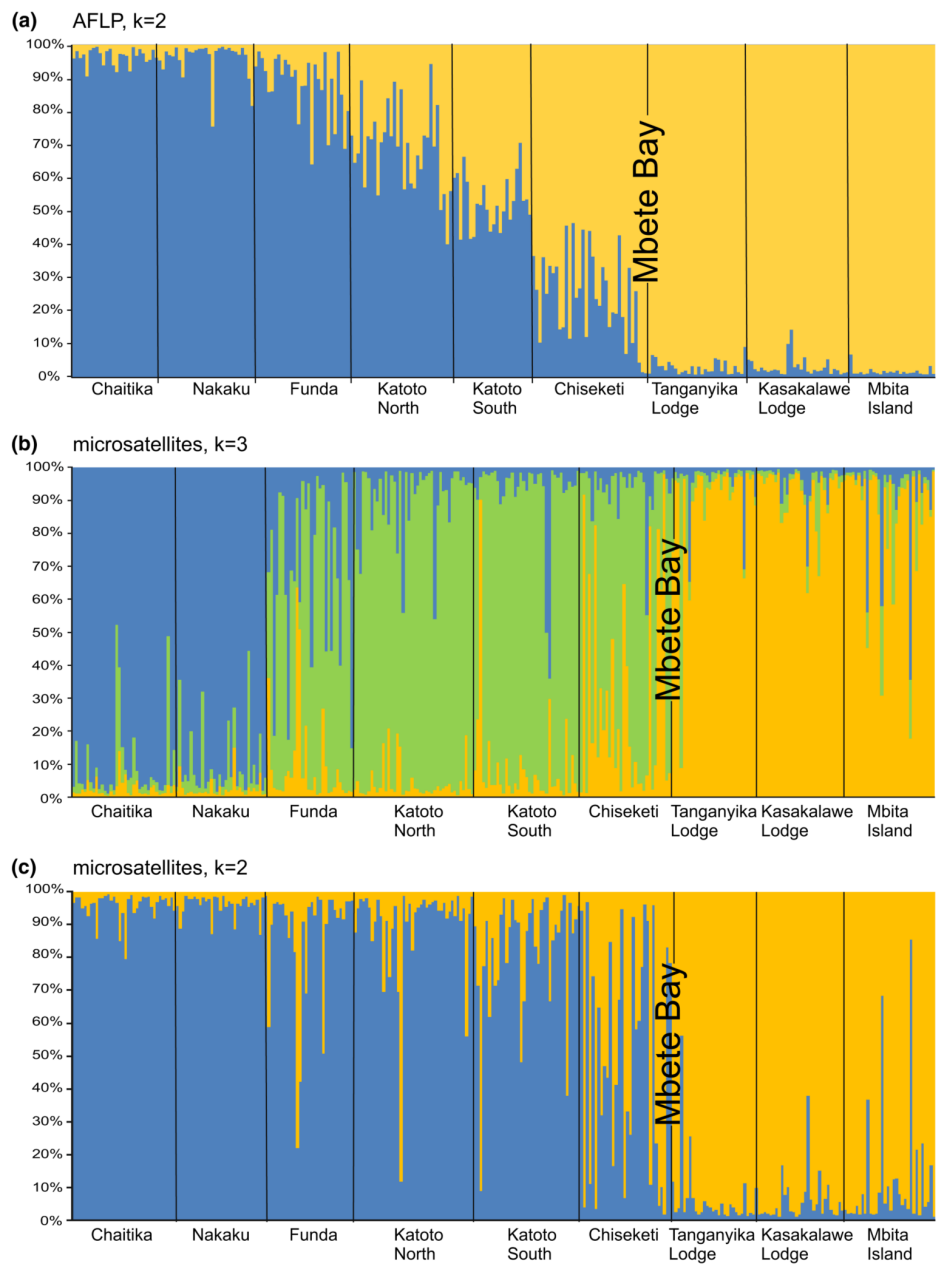
Bayesian cluster analysis (STRUCTURE) of the investigated *Tropheus moorii* populations. Each vertical bar corresponds to one individual and colored sections of each bar represent the individual’s assignment probability to the reconstructed genetic clusters. Populations are separated by black lines. **a** Assignments based on AFLP genotypes for the most likely solution of *K* = 2 clusters. **b** Assignments based on microsatellite genotypes for the most likely solution of *K* = 3 clusters. **c** Assignments into *K* = 2 clusters based on microsatellite genotypes

Patterns of admixture in nuclear markers were congruent with the mitochondrial data and likewise indicated unidirectional gene flow across Mbete Bay. The STRUCTURE analysis of the AFLP dataset returned a most likely number of two genetic clusters, corresponding to one bluish and one yellow-blotch clade, with an admixture cline in those populations west of Mbete Bay that contained both mitochondrial lineages (Fig. 4a; Online Resource 1). With microsatellite data, STRUCTURE estimated a most likely number of three genetic clusters (Fig. 4b; Online Resource 1). One cluster represented the two bluish populations most distant from Mbete Bay, which contained only bluish mtDNA haplotypes; the second cluster corresponded to the populations west of Mbete Bay that showed mitochondrial admixture; and the third cluster consisted of the yellow-blotch populations east of Mbete Bay. The Bayesian reconstruction of three genetic clusters from microsatellites contrasts with the two clusters recognized in the AFLP data. Possibly, codominance and the higher mutation rates of microsatellites allow improved detection of genetic structure by the STRUCTURE approach. A STRUCTURE analysis of microsatellite data constrained to *K* = 2 revealed an admixture cline that was congruent with the pattern observed with mtDNA and AFLP data (Figs. 3a, 4c).

Nuclear admixture per population was quantified as the average per-individual assignment probability to the bluish lineage (*q* values of the STRUCTURE analysis). Nuclear and mtDNA markers consistently reflected a cline in admixture between bluish and yellow-blotch *Tropheus* genomes in the populations west of Mbete Bay and an abrupt drop in admixture proportions in the populations east of the bay (Fig. 3a).

### Diversity and population structure

Both mitochondrial and nuclear markers displayed a high level of genetic diversity within and differentiation among populations (Table 1; Online Resource 2). Overall estimates of population differentiation were *F*_ST_ = 0.059 for AFLP, *F*_ST_ = 0.029 for microsatellite, *F*_ST_ = 0.034 and *ϕ*_ST_ = 0.50 for mtDNA markers, all with *P* < 0.001. Pairwise population differentiation (Online Resource 2) was distinct and significant, except for weak or no differentiation across all three marker types between Chaitika and Nakaku as well as between Tanganyika Lodge and Kasakalawe Lodge; in both cases, sampling locations are connected by continuous rocky habitat. Additionally, mitochondrial differentiation estimated by *ϕ*_ST_ was non-significant in three comparisons among adjacent populations (Katoto North—Funda, Katoto South—Chiseketi, Kasakalawe Lodge—Mbita) and narrowly missed significance based on *F*_ST_ estimates in three other population pairs (Katoto South— Kasakalawe Lodge, *P* = 0.06; Katoto South—Tanganyika Lodge, *P* = 0.06; Katoto North—Chiseketi, *P* = 0.13).

### Demographic reconstruction of the admixture event

**Fig. 5:**
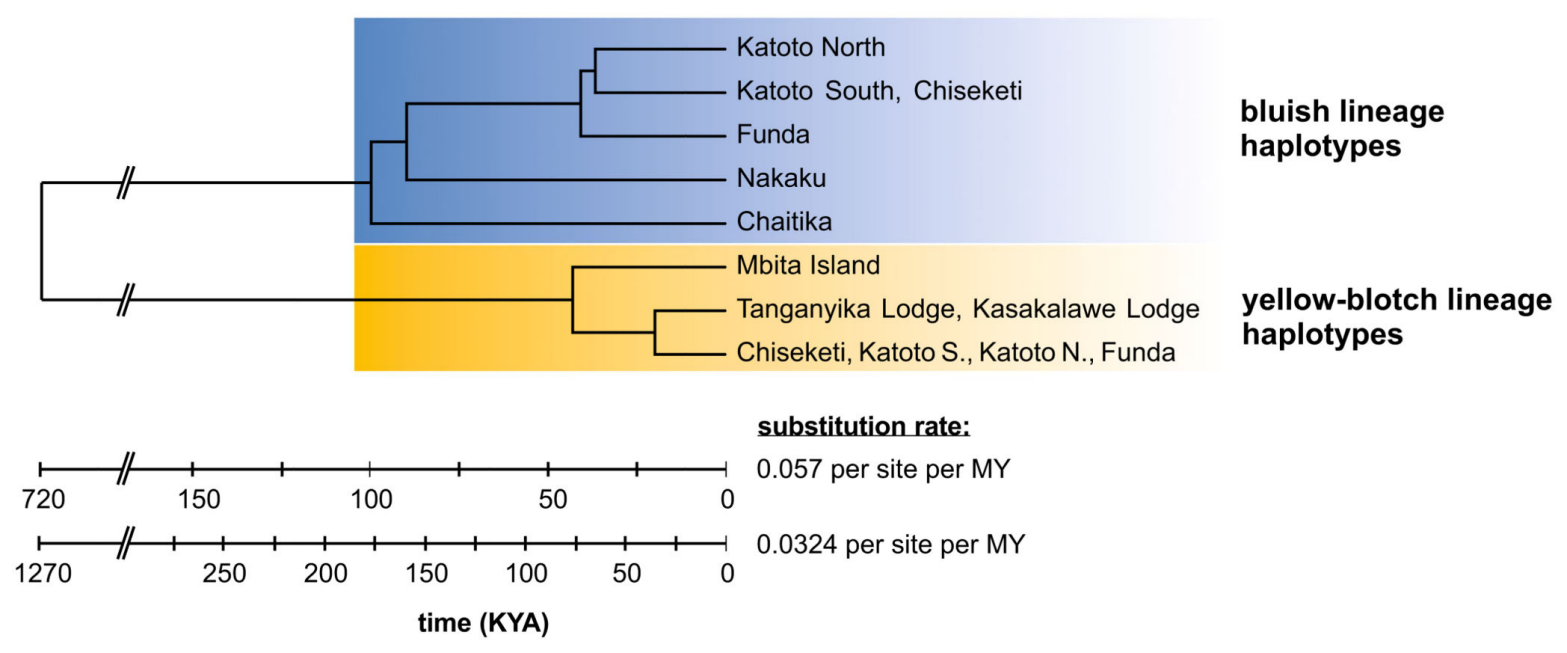
Chronology of population splitting reconstructed from mtDNA data. Samples from admixed populations were grouped by haplotype lineage. Pairwise population divergence was modeled in IMa2; splitting time between the two major lineages was estimated as net divergence in MEGA

**Fig. 6:**
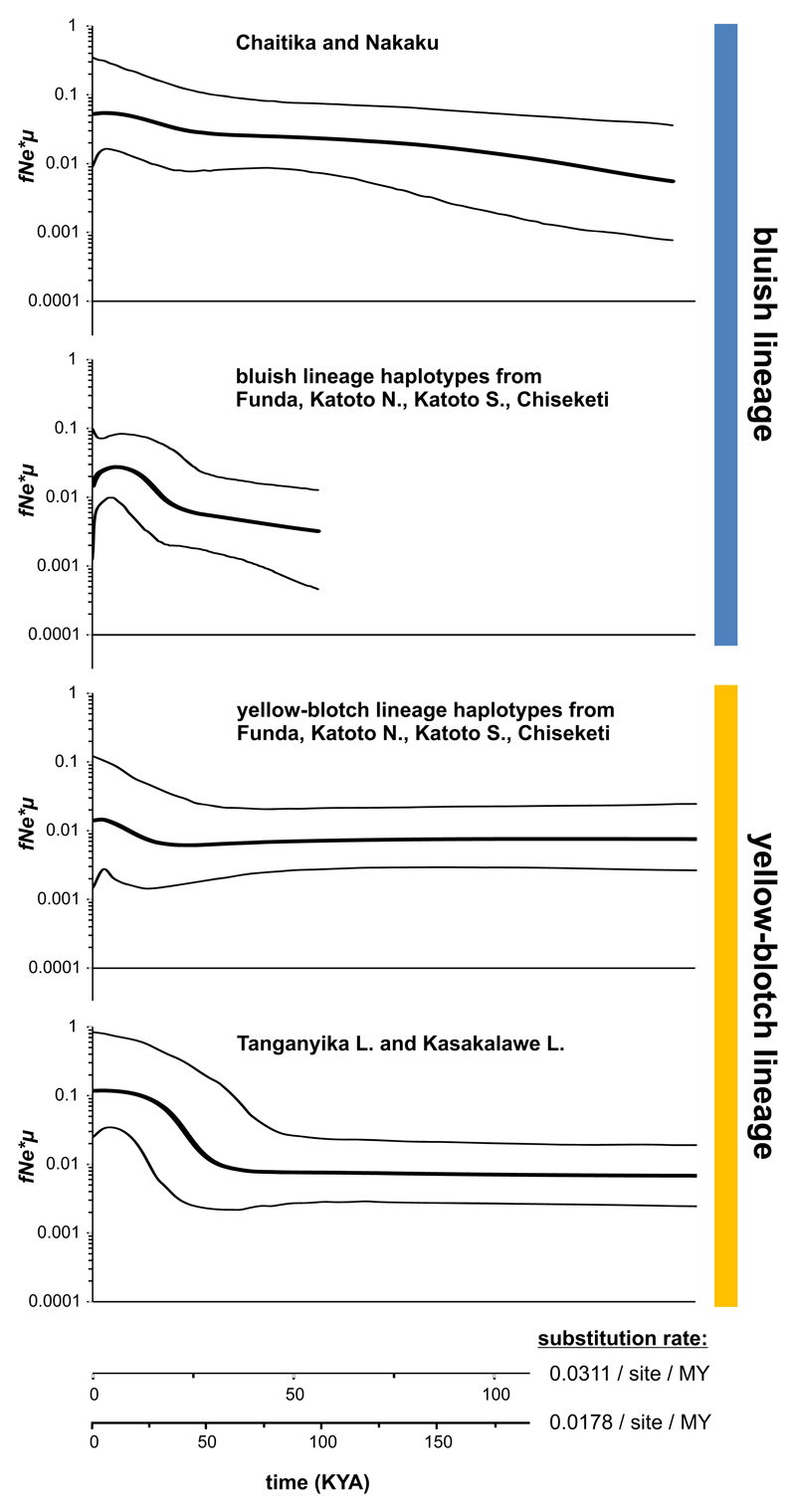
Bayesian skyline plots of population sizes through time. *Thick* and *thin lines* represent the median and 95% HPD intervals, respectively. The *y*-axis represents the population size parameter (female effective population size times the mutation rate)

In the following, “admixed populations” refers to the populations containing haplotypes from both mtDNA lineages (Chiseketi, Katoto North, Katoto South, Funda), as opposed to “pure bluish populations” (Nakaku and Chaitika) and “pure yellow-blotch populations” (Kasakalawe Lodge, Tanganyika Lodge and Mbita; Figs. 2, 3a). Linkage disequilibrium among nuclear loci was not elevated in admixed relative to pure populations (Fig. 3b), and many of the “yellow-blotch lineage” haplotypes found in the admixed populations were not shared with the pure yellow-blotch populations (Fig. 2). These observations and the fact that the stenotopic ecology of *Tropheus* makes high rates of migration across the 6 km wide stretch of sand unlikely suggest that the major burst of introgression occurred at some point in the past, when the habitat structure in the area of Mbete Bay allowed migration. IMa2 dated the split of “yellow-blotch lineage” haplotypes found in the admixed populations from the two closest pure yellow-blotch populations (Kasakalawe and Tan-ganyika Lodge) to a divergence time parameter of 0.489 (mean of four IMa2 runs). Higher divergence time parameter estimates (mean estimates of 0.905–1.016) were obtained between the “bluish lineage” haplotype groups from the different admixed locations west of Mbete Bay (Fig. 5; Online Resource 3). This implies that the area occupied by the admixed populations was first colonized by bluish lineage *Tropheus* when the habitat became available after a lake lowstand, and then introgressed by yellow-blotch-lineage *Tropheus* migrating from the east. Reconstructions of population sizes through time indicated recent rapid expansions of the pure yellow-blotch populations east of Mbete Bay and of the “yellow-blotch lineage” haplotypes in the admixed populations (Fig. 6). In contrast, the pure bluish populations and the “bluish lineage” haplotypes in the admixed populations expanded over a longer period of time, and the “bluish lineage” haplotypes in the admixed populations experienced a recent decline that coincided with growth of the “yellow-blotch lineage” haplotypes in these populations (Fig. 6).

## Discussion

### Congruent patterns of mitochondrial and nuclear admixture reject link between mate choice and introgression asymmetry

Congruent unidirectional nuclear and mitochondrial introgression across Mbete Bay rejects the hypothesis that *Tropheus* migrated in both directions across the bay and mate choice behavior mediated nuclear but not mitochondrial introgression into the yellow-blotch populations east of the bay. Rather, genetic admixture between yellow-blotch and bluish lineages was confined to the locations west of Mbete Bay, where the genetic contribution of the yellow-blotch lineage declined with distance from Mbete Bay. At Mbete Bay, the admixture cline ends with a sharp drop in the proportion of genetic material assigned to the bluish lineage (Fig. 3a). Current migration of the stenotopic rock-dweller across the wide sand barrier is unlikely given that much smaller habitat barriers curb gene flow in this species (Koblmüller et al., 2011; current study), but the genetic data are consistent with a scenario of past migration facilitated by transiently favorable habitat conditions.

### An alternative scenario: asymmetric admixture cline shaped by habitat changes

Having rejected asymmetric mate choice as a mechanism causing unidirectional introgression, the question remains why introgression across Mbete Bay occurred in one direction only. A possible explanation involves changes of habitat structure and the population displacements that occurred during lake level fluctuations, which particularly affected the littoral communities of Lake Tanganyika (Cohen et al., 2007; McGlue et al., 2008). Specifically, at the time of secondary contact between the two *Tropheus* lineages, the area of the presently sandy Mbete Bay may have looked differently and perhaps provided suitable habitat for a westward expansion of yellow-blotch *Tropheus*. The divergence time estimates in the present study coincide with estimates for splits among yellow-blotch populations between Mbete and Chituta Bay (Koblmüller et al., 2011), which were attributed to the colonization of the area after the lake level had been reduced by ~260 m during the last glacial maximum at around 30,000 to 15,000 years BP (Cohen et al., 1997; McGlue et al., 2008). In the present as well as in the previous study (Fig. 5; Koblmüller et al., 2011), the estimated population divergence times are older than the paleolimnological dating of the lake level rise. This is expected because substitution rate estimates that are based on an ancient event (in our case, ~1MYA; Nevado et al., 2009) are bound to overestimate the age of recent splits (Ho et al., 2005). With the southward expansion of the lake’s shoreline that accompanied the rising lake level (Fig. 1; McGlue et al., 2008), bluish *Tropheus* colonized the shoreline west of Mbete Bay from their northern retreat, whereas yellow-blotch *Tropheus* moved into the area east of Mbete Bay from the opposite direction. Habitat structure during the lake level rise may have allowed yellow-blotch *Tropheus* to expand their range along rocky habitat until they came into contact with the bluish *Tropheus*. After an admixture zone was formed by introgression between the two lineages, progressing sedimentation of Mbete Bay may then have caused local extinctions of *Tropheus* populations, which may have truncated the eastern section of the admixture cline and shaped the current asymmetric pattern. This scenario is contingent on the transient inhabitability of the Mbete Bay area for a rock-dwelling fish, which is not altogether unlikely given that a change in the lake level is expected to redistribute the sediment and alter the sedimentation system (Andy Cohen, pers. comm.). Although Mbete Bay represents a divide between genetic and phenotypic clusters for several cichlid species (Kohda et al., 1996; Duftner et al., 2006; Koblmüller et al., 2007; Sefc et al., 2007; Koblmüller et al., 2009), haplotype networks of some rock-dwelling species show signatures of intro-gression between the genetic lineages found east and west of bay (*Perissodus microlepis*: Koblmüller et al., 2009; *Variabilichromis moorii*: Duftner et al., 2006; *Eretmodus cyanostictus*, *Ophthalmotilapia ventralis*: Sefc et al., 2007), although never to that extent as detected in *Tropheus moorii*.

### Demographic expansion of yellow-blotch lineage haplotypes in the admixed populations

The demographic expansion of the “yellow-blotch lineage” haplotype group and simultaneous decline of the “bluish lineage” clade in the admixed populations suggest that reproductive success of females carrying the yellow-blotch haplotype exceeded that of females carrying the bluish haplotype. Selection favoring one haplotype lineage may have a physiological underpinning, for instance, associated with energy metabolism (Shen et al., 2010), but could also be related to the asymmetric mate preferences that were initially hypothesized to drive unidirectional mitochondrial introgression. During admixture, a declining mating propensity of the discriminating bluish females in the face of an increasing frequency of intermediate *Tropheus* phenotypes would have been contrasted with ready reproduction by yellow-blotch females, which could have caused decline and expansion, respectively, of the two mitochondrial lineages. Finally, lineage-specific differences in female fecundity could also produce the observed pattern.

## Conclusion

Patterns of unidirectional introgression between species or geographically separated clades are frequently observed in genetic studies, but the mechanisms behind the asymmetry are seldom inferred conclusively (Zhang, 2014). Exceptions are cases that involve geographic barriers that can clearly be negotiated in only one direction, such as waterfalls or water currents for aquatic organisms (Papetti et al., 2012; Schilthuizen et al., 2012; Schenekar et al., 2014; Reis et al., 2015). In the absence of such external sources of asymmetry, suggested causes for asymmetry typically include mate choice (Nevado et al., 2011; Pons et al., 2014) and cytonuclear incompatibilities (Arntzen et al., 2009; Nevado et al., 2011). In the present example, the pattern of unidirectional introgression can be explained on the basis of habitat changes, which first facilitated the development of a symmetric admixture zone and then shaped asymmetry by causing local extinctions and cessation of gene flow. Habitat structure and dynamics have already previously been suggested to be important factors in the evolution and diversification of *Tropheus* (Egger et al., 2007, 2010; Koblmüller et al., 2011; Sefc et al., 2015; Van Steenberge et al., 2015), and the present study supports this by once again implying habitat structure as a primary determinant of population structure and introgression patterns. In this respect, *Tropheus* may be representative of littoral rock dwellers, or habitat specialists, that find themselves at the mercy of the dynamics of their preferred habitat.

## Supplementary Material

**Electronic supplementary material** The online version of this article (doi:10.1007/s10750-016-2791-x) contains supplementary material, which is available to authorized users.

Suppl material 3

Suppl material 2

Suppl material 1

## Figures and Tables

**Table 1:** Population samples, sample sizes (*n*), and genetic diversity at mtDNA, AFLP and microsatellite markers.

Population	Color pattern type	mtDNA			AFLP			Microsatellites		
		*n*	*h*	*H*_E_	*n*	*PLP* (%)	*H*_E_	*n*	*NA*	*H*_E_
*West of Mbete Bay*										
Chaitika	Bluish	32	24	0.970	24	37.2	0.125	36	18.25	0.846
Nakaku	Bluish	32	20	0.962	30	40.3	0.124	32	16.44	0.838
Funda	Bluish	34	23	0.970	28	38.5	0.123	30	15.31	0.842
Katoto North	Bluish	36	23	0.935	29	38.8	0.121	44	16.75	0.842
Katoto South	Intermediate	32	19	0.960	28	38.9	0.122	36	16.06	0.840
Chiseketi	Intermediate	40	17	0.935	32	35.5	0.112	32	15.38	0.851
*East of Mbete Bay*										
Tanganyika Lodge	Yellow-blotch	28	21	0.942	31	37.7	0.109	30	14.69	0.816
Kasakalawe Lodge	Yellow-blotch	30	23	0.968	30	38.9	0.118	30	15.00	0.835
Mbita	Yellow-blotch	31	23	0.974	29	36.5	0.107	31	14.75	0.818

*h* number of different haplotypes; *H*_E_ expected heterozygosity; *PLP* proportion of polymorphic loci (frequency of less frequent allele ≥5%); *NA* number of alleles
