# Supplementary material for: Past lake shore dynamics explain present pattern of unidirectional introgression across a habitat barrier
Source: Hydrobiologia. Author manuscript; Available in PMC 2019 Jun 10. (PMC6557712; doi:10.1007/s10750-016-2791-x)
Supplement: Suppl material 3 [file EMS83186-supplement-Suppl_material_3.pdf]

Online Resource 3 Ima2 maximum likelihood estimates of the parameters for population divergence times (*t*), the effective population sizes of the ancestral (*q2*) and the daughter populations (*q0*, *q1*) and migration rates (*m0>1*, *m1>0*). Samples from admixed populations were grouped by haplotype lineage. Lists of populations separated by / indicate pooled samples. Values in parentheses represent the interval of the 90% highest posterior density (HPD).

**population 0 versus population 1**	***t***	***q0***	***q1***	***q2***	***m0>1***	***m1>0***
**Bluish-lineage mtDNA haplotypes**						
Chiseketi/Katoto/Funda versus Nakaku/Chaitika, run 1	2.308 (1.423 - 3.748)	114.3 (77.75 - 169.8)	130.8 (81.75 - 215.3)	16.75 (2.75 - 52.25)	0.05 (0 - 0.27)	0.07 (0 - 0.31)
Chiseketi/Katoto/Funda versus Nakaku/Chaitika, run 2	2.293 (1.418 - 3.735)	113.3 (77.25 - 168.8)	131.8 (81.75 - 214.8)	17.25 (2.75 - 51.75)	0.06 (0 - 0.42)	0.06 (0 - 0.5)
Chiseketi/Katoto/Funda versus Nakaku/Chaitika, run 3	2.293 (1.432 - 3.741)	114.3 (77.25 - 169.8)	131.3 (81.75 - 214.8)	17.25 (2.75 - 52.25)	0.05 (0 - 0.27)	0.07 (0 - 0.33)
Chiseketi/Katoto South versus Funda, run 1	1.065 (0.609 - 1.999)	31.75 (10.75 - 79.75)	95.25 (41.25 - 400.3)	14.25 (2.25 - 50.75)	0.49 (0 - 1.85)	0.01 (0 - 0.75)
Chiseketi/Katoto South versus Funda, run 2	1.087 (0.4815 - 2.723)	31.5 (10.5 - 77.5)	88.5 (31.5 - 513.5)	10.5 (1.5 - 49.5)	0.51 (0 - 1.87)	0.01 (0 - 0.79)
Chiseketi/Katoto South versus Katoto North, run 1	0.8745 (0.5865 - 2.998)	29.5 (9.5 - 70.5)	98.5 (30.5 - 602.5)	24.5 (1.5 - 67.5)	0.37 (0 - 2.15)	0.01 (0 - 2.53)
Chiseketi_Katoto South versus Katoto North, run 2	0.9345 (0.6045 - 2.998)	29.5 (9.5 - 70.5)	97.5 (30.5 - 589.5)	23.5 (1.5 - 66.5)	0.35 (0 - 2.17)	0.01 (0 - 2.57)
Cihiseketi versus Funda, run 1	1.059 (0.539 - 1.999)	10.75 (2.25 - 41.25)	110.8 (47.75 - 422.8)	16.75 (2.75 - 53.75)	0.8737 (0 - 2.33)	0.01 (0 - 0.83)
Cihiseketi versus Funda, run 2	0.9645 (0.4515 - 2.921)	10.5 (2.4 - 50.5)	96.5 (35.5 - 614.5)	12.5 (0 - 53.5)	0.39 (0 - 2.41)	0.01 (0 - 1.15)
Katoto South versus Funda, run 1	0.955 (0.539 - 1.783)	36.25 (7.25 - 152.3)	119.3 (48.25 - 449.3)	12.75 (2.75 - 38.25)	0.11 (0 - 1.75)	0.01 (0 - 0.95)
Katoto South versus Funda, run 2	0.9135 (0.4755 - 2.015)	35.5 (7.5 - 149.5)	100.5 (34.5 - 723.5)	12.5 (2.5 - 39.5)	0.11 (0 - 1.81)	0.01 (0 - 0.97)
Katoto North versus Funda, run 1	1.004 (0.5295 - 2.209)	82.5 (20.5 - 297.5)	105.5 (20.5 - 624.5)	18.5 (3.5 - 49.5)	0.01 (0 - 1.87)	0.07 (0 - 1.41)
Katoto North versus Funda, run 2	0.9555 (0.5295 - 2.2)	83.5 (20.5 - 294.5)	107.5 (31.5 - 620.5)	18.5 (3.5 - 49.5)	0.01 (0 - 1.83)	0.07 (0 - 1.43)

Chiseketi/Katoto/Funda versus Chaitika, run 1	2.606 (1.591 - 2.998)	113.5 (74.5 - 171.5)	107.5 (52.5 - 235.5)	17.5 (4.5 - 57.5)	0.05 (0 - 0.27)	0.01 (0 - 0.31)
Chiseketi/Katoto/Funda versus Chaitika, run 2	2.553 (1.278 - 4.598)	111.5 (71.5 - 172.5)	101.5 (48.5 - 229.5)	14.5 (1.5 - 59.5)	0.05 (0 - 0.29)	0.01 (0 - 0.33)
Chiseketi/Katoto/Funda versus Nakaku, run 1	2.171 (1.333 - 2.998)	129.5 (85.5 - 200.5)	59.5 (27.5 - 117.5)	19.5 (3.5 - 58.5)	0.01 (0 - 0.25)	0.15 (0 - 0.57)
Chiseketi/Katoto/Funda versus Nakaku, run 2	2.268 (1.087 - 3.928)	126.5 (84.5 - 197.5)	58.5 (28.5 - 116.5)	16.5 (1.5 - 55.5)	0.03 (0 - 0.25)	0.15 (0 - 0.57)
Nakaku versus Chaitika, run 1	2.288 (0.6735 - 2.998)	63.5 (12.5 - 185.5)	120.5 (40.5 - 801.5)	20.5 (4.5 - 78.5)	0.03 (0 - 2.07)	0.73 (0 - 2.47)
Nakaku versus Chaitika, run 2	2.293 (0.4725 - 4.497)	61.5 (13.5 - 169.5)	111.5 (38.5 - 806.5)	16.5 (0 - 74.5)	0.03 (0 - 2.03)	0.81 (0 - 2.63)
**Yellow-blotch lineage mtDNA haplotypes**						
Mbita versus Chiseketi/Katoto/Funda, run 1	1.256 (0.6645 - 2.998)	110 (38 - 322)	38 (14 - 94)	46 (18 - 110)	0.17 (0 - 1.05)	0.01 (0 - 0.91)
Mbita versus Chiseketi/Katoto/Funda, run 1	1.222 (0.6705 - 2.909)	110 (38 - 326)	38 (14 - 94)	46 (18 - 110)	0.17 (0 - 1.05)	0.01 (0 - 0.91)
Tanganyika Lodge/Kasakalawe Lodge versus Mbita, run 1	0.9165 (0.4665 - 1.7)	152.5 (69.5 - 654.5)	113.5 (36.5 - 345.5)	44.5 (19.5 - 83.5)	0.01 (9 - 0.53)	0.13 (0 - 1.23)
Tanganyika Lodge/Kasakalawe Lodge versus Mbita, run 2	0.8715 (0.4605 - 1.690)	153.5 (69.5 - 665.5)	13.5 (39.5 - 343.5)	44.5 (19.5 - 83.5)	0.01 (0 - 0.53)	0.13 (0 - 1.17)
Tanganyika Lodge/Kasakalawe Lodge versus Chiseketi/Katoto/Funda, run 1	0.4995 (0.2835 - 2.998)	578 (258 - 3822)	30 (10 - 98)	42 (14 - 110)	1.93 (0 - 3.25)	0.17 (0 - 1.87)
Tanganyika Lodge/Kasakalawe Lodge versus Chiseketi/Katoto/Funda, run 2	0.4755 (0.2895 - 2.998)	582 (258 - 3826)	30 (10 - 98)	42 (14 - 110)	1.95 (0 - 3.27)	0.17 (0 - 1.85)
Tanganyika Lodge/Kasakalawe Lodge versus Chiseketi/Katoto/Funda, run 3	0.4965 (0.2685 - 2.998)	578 (250 - 3806)	30 (10 - 102)	42 (14 - 110)	1.89 (0 - 3.25)	0.15 (0 - 1.99)
Tanganyika Lodge/Kasakalawe Lodge versus Chiseketi/Katoto/Funda, run 4	0.4845 (0.2835 - 2.998)	574 (258 - 3822)	30 (10 - 98)	42 (14 - 110)	1.95 (0 - 3.29)	0.17 (0 - 1.89)
Mbita/Tanganyika Lodge/Kasakalawe Lodge versus Chiseketi/Katoto/Funda, run 1	0.8415 (0.3825 - 2.981)	394 (166 - 3218)	34 (14 - 90)	46 (14 - 110)	1.23 (0.05 - 2.69)	0.13 (0 - 1.53)
Mbita/Tanganyika Lodge/Kasakalawe Lodge versus Chiseketi/Katoto/Funda, run 2	0.6555 (0.3405 - 2.998)	414 (166 - 3226)	34 (14 - 90)	50 (18 - 114)	1.35 (0 - 2.59)	0.15 (0 - 1.67)
