# Supplementary material for: Past lake shore dynamics explain present pattern of unidirectional introgression across a habitat barrier
Source: Hydrobiologia. Author manuscript; Available in PMC 2019 Jun 10. (PMC6557712; doi:10.1007/s10750-016-2791-x)
Supplement: Suppl material 2 [file EMS83186-supplement-Suppl_material_2.pdf]

Table S1 Pair-wise mitochondrial population differentiation between the investigated *T. moorii* populations calculated with ARLEQUIN v.3.1. F_{ST} values are below diagonal, Φ_{ST} values above. Benjamini-Hochberg corrected significance levels: *** p < 0.001; ** p < 0.01; * p < 0.05

	Chaitika	Nakaku	Funda	KatN	KatS	Chiseketi	TanL	KasL	Mbita
Chai		0.047*	0.389***	0.288***	0.291***	0.282***	0.781***	0.776***	0.758***
Naka	0.018*		0.293***	0.212***	0.244***	0.242***	0.762***	0.758***	0.740***
Fund	0.026***	0.033***		0.031	0.160***	0.200***	0.786***	0.780***	0.761***
KatN	0.040***	0.050***	0.044***		0.081*	0.122**	0.705***	0.702***	0.687***
KatS	0.031***	0.038***	0.034***	0.016*		-0.012	0.478***	0.476***	0.466***
Chis	0.044***	0.051***	0.045***	0.009	0.019*		0.407***	0.406***	0.400***
TanL	0.040***	0.048***	0.044***	0.060***	0.016	0.052***		0.001	0.077**
KasL	0.027***	0.035***	0.031***	0.047***	0.014	0.036***	-0.003		0.022
Mbit	0.024***	0.032***	0.028***	0.045***	0.033***	0.042***	0.037***	0.015*	

Chai = Chaitika; Naka = Nakaku; Fund = Funda; KatN = Katoto North; KatS = Katoto South; Chis = Chiseketi; TanL = Tanganyika Lodge; KasL = Kasakalawe Lodge; Mbit = Mbita Island

Table S2 Pair-wise genetic differentiation in AFLP alleles between the nine investigated populations calculated with AFLP-SURV v.1.0. F_{ST} values are in the bottom left corner, corresponding *P*-values in upper right corner. Calculation of *P*-values was based on 5000 permutations

	Chai	Naka	Fund	KatN	KatS	Chis	TanL	KasL	Mbit
Chai		<0.0001	<0.0001	<0.0001	<0.0001	<0.0001	<0.0001	<0.0001	<0.0001
Naka	0.0079		<0.0001	<0.0001	<0.0001	<0.0001	<0.0001	<0.0001	<0.0001
Fund	0.0321	0.0318		<0.0001	<0.0001	<0.0001	<0.0001	<0.0001	<0.0001
KatN	0.0545	0.0577	0.0215		<0.0001	<0.0001	<0.0001	<0.0001	<0.0001
KatS	0.0536	0.0563	0.0209	0.0055		<0.0001	<0.0001	<0.0001	<0.0001
Chis	0.0730	0.0798	0.0409	0.0491	0.0284		<0.0001	<0.0001	<0.0001
TanL	0.1009	0.1082	0.0762	0.0753	0.0465	0.0224		<0.0001	<0.0001
KasL	0.1048	0.1078	0.0781	0.0616	0.0422	0.0321	0.0118		<0.0001
Mbit	0.1138	0.1215	0.0951	0.0998	0.0676	0.0461	0.0246	0.0371	

Chai = Chaitika; Naka = Nakaku; Fund = Funda; KatN = Katoto North; KatS = Katoto South; Chis = Chiseketi; TanL = Tanganyika Lodge; KasL = Kasakalawe Lodge; Mbit = Mbita Island

Table S3 Pair-wise population differentiation (F_{ST}) between the nine investigated *T. moorii* populations based on 16 microsatellite loci. Benjamini-Hochberg corrected significance levels: *** p < 0.001; ** p < 0.01; * p < 0.05

	Chaitika	Nakaku	Funda	KatN	KatS	Chiseketi	TanL	KasL	Mbita
Chaitika									
Nakaku	0.007**								
Funda	0.016***	0.023***							
Katoto N	0.024***	0.030***	0.013***						
Katoto S	0.022***	0.030***	0.008***	0.007***					
Chiseketi	0.025***	0.035***	0.018***	0.014***	0.012***				
TanL	0.048***	0.064***	0.047***	0.051***	0.037***	0.017***			
KasL	0.039***	0.049***	0.042***	0.043***	0.032***	0.014***	0.001		
Mbita	0.039***	0.055***	0.046***	0.048***	0.038***	0.025***	0.017***	0.008***	

Katoto N = Katoto North; Katoto S = Katoto South; TanL = Tanganyika Lodge; KasL = Kasakalawe Lodge; Mbita = Mbita Island
