# Supplementary material for: Past lake shore dynamics explain present pattern of unidirectional introgression across a habitat barrier
Source: Hydrobiologia. Author manuscript; Available in PMC 2019 Jun 10. (PMC6557712; doi:10.1007/s10750-016-2791-x)
Supplement: Suppl material 1 [file EMS83186-supplement-Suppl_material_1.pdf]

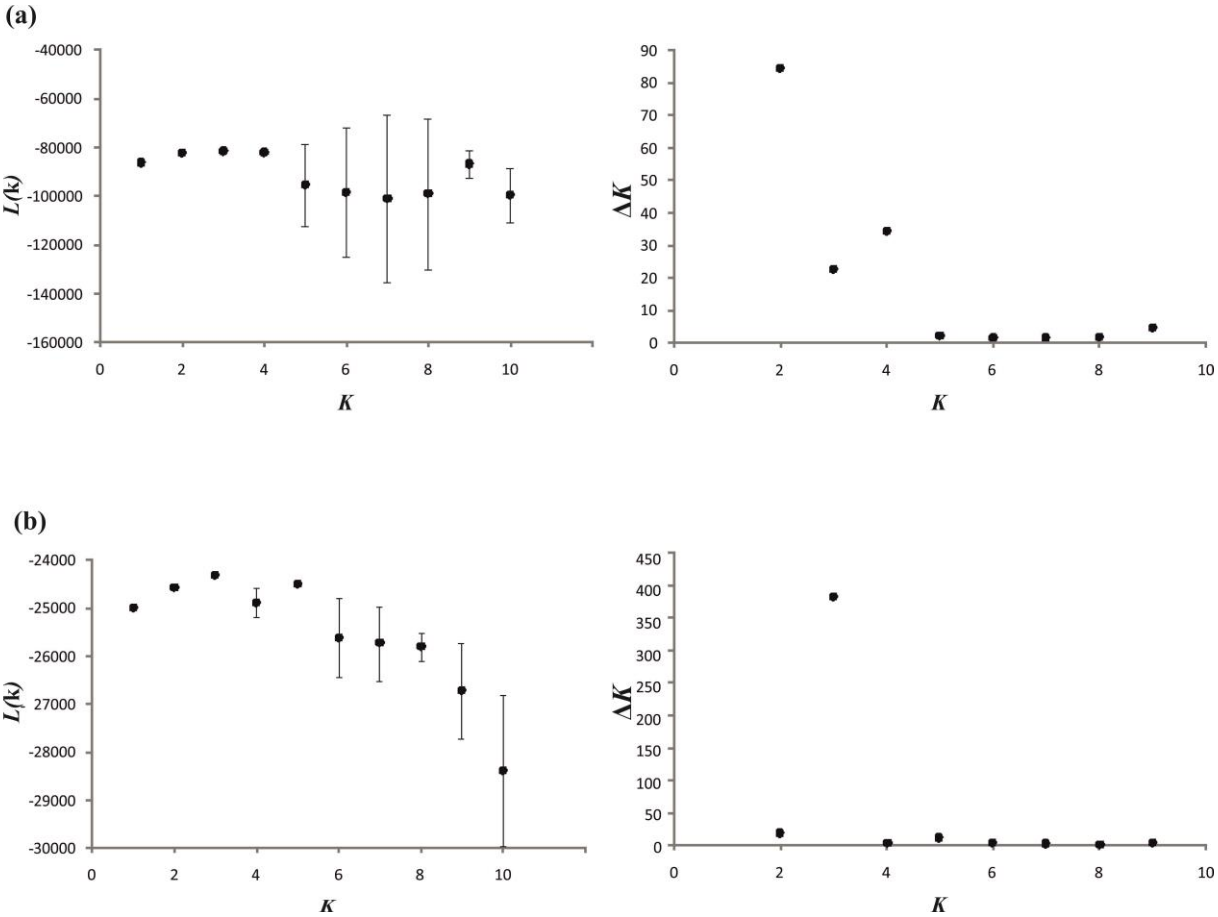

Online Resource 1. Log likelihood values and corresponding Δ*K* values (as described in Evanno et al., 2005) for all *K*'s (number of genetic clusters) tested, for a) the AFLP dataset and b) the SSR dataset.
